# Methods and initial characteristics of aged participants in a randomized clinical trial on cognitive stimulation

**DOI:** 10.1002/alz.090062

**Published:** 2025-01-03

**Authors:** Thais Bento Lima Silva, Tiago Nascimento Ordonez, Gabriela dos Santos, Ana Paula Bagli Moreira, Laydiane Alves Costa, Bárbara Perpétuo, Patrícia Prata Lessa, Luiz Carlos Moraes, Neide Pereira Cardoso, Sonia Brucki, Monica Sanches Yassuda

**Affiliations:** ^1^ Cognitive and Behavioral Neurology Unit (GNCC) of the University of São Paulo., São Paulo, São Paulo Brazil; ^2^ University of São Paulo, São Paulo Brazil; ^3^ University of São Paulo, São Paulo, São Paulo Brazil; ^4^ Universidade de São Paulo (USP), São Paulo, São Paulo Brazil; ^5^ USP, São Paulo, Brazil Brazil; ^6^ Universidade de São Paulo, São Paulo, São Paulo Brazil; ^7^ Supera Institute of Education, São José dos Campos, São Paulo Brazil; ^8^ Universidade de São Paulo ‐ USP, São Paulo Brazil; ^9^ University of São Paulo Medical School, São Paulo Brazil; ^10^ University of São Paulo, SAO PAULO Brazil; ^11^ University of São Paulo, SAO PAULO, SAO PAULO Brazil; ^12^ Medical School of University of São Paulo, São Paulo Brazil; ^13^ State University of Campinas UNICAMP, Campinas Brazil; ^14^ Universidade de São Paulo, São Paulo Brazil

## Abstract

**Background:**

Scientific investigations underscore the cognitive, psychological, and social benefits of cognitive stimulation for aged individuals, yet rigorous long‐term studies remain limited.

**Method:**

This abstract outlines the methods and initial participant characteristics of a controlled, randomized clinical trial on cognitive stimulation. A total of 578 aged individuals responded to the study call, with 362 meeting eligibility criteria. Subsequently, 255 participants were selected and randomized into Training Group (TG), Active Control Group (ACG), and Passive Control Group (PCG). During the baseline phase (T0), 48 participants withdrew, resulting in a T0 sample of 207 participants.

**Result:**

The three groups exhibited homogeneity in cognitive performance, sociodemographic, and psychosocial variables, with only depressive symptoms showing a significant difference, where TG scored higher. Despite this difference, other variables indicated equivalence among the research groups.

**Conclusion:**

The outlined methods serve as inspiration for future research on cognitive stimulation in the aged.

